# Methyl (2*Z*)-2-{(2*Z*)-3-[(cyclo­pentyl­idene)amino]-4-oxo-2-phenyl­imino-1,3-thia­zol­idin-5-yl­idene}acetate

**DOI:** 10.1107/S1600536814004048

**Published:** 2014-02-26

**Authors:** Joel T. Mague, Mehmet Akkurt, Shaaban K. Mohamed, Alaa A. Hassan, Mustafa R. Albayati

**Affiliations:** a Department of Chemistry, Tulane University, New Orleans, LA 70118, USA; b Department of Physics, Faculty of Sciences, Erciyes University, 38039 Kayseri, Turkey; c Chemistry and Environmental Division, Manchester Metropolitan University, Manchester M1 5GD, England; d Chemistry Department, Faculty of Science, Minia University, 61519 El-Minia, Egypt; e Kirkuk University, College of Science, Department of Chemistry, Kirkuk, Iraq

## Abstract

In the title compound, C_17_H_17_N_3_O_3_S, the thia­zole ring is nearly planar [maximum deviation = 0.015 (1) Å for the ring N atom] and the cyclo­pentane ring has a twist conformation. The mol­ecular conformation is stabilized by a hypervalent inter­action between the S atom and the ester group carbonyl O atom, with an S⋯O distance of 2.7931 (10) Å. In the crystal, C—H⋯O inter­actions generate chains of mol­ecules propagating along [110] and π–π stacking inter­actions [centroid–centroid distance = 3.4677 (7) Å] between the thia­zole rings organize these chains into (001) layers.

## Related literature   

For the synthesis and similar structures, see: Akkurt *et al.* (2009 ▶); Li *et al.* (2011 ▶); Mague *et al.* (2013 ▶); Mohamed *et al.* (2013*a* ▶,*b* ▶); Pomés Hernández *et al.* (1996 ▶); Sundar *et al.* (2003 ▶). For the general biological significance of thia­zolidinone scaffold compounds, see: Pfützner *et al.* (2007 ▶); Schianca *et al.* (2012 ▶); Jain *et al.* (2012 ▶); Lant (1986 ▶); Rock *et al.* (1991 ▶).
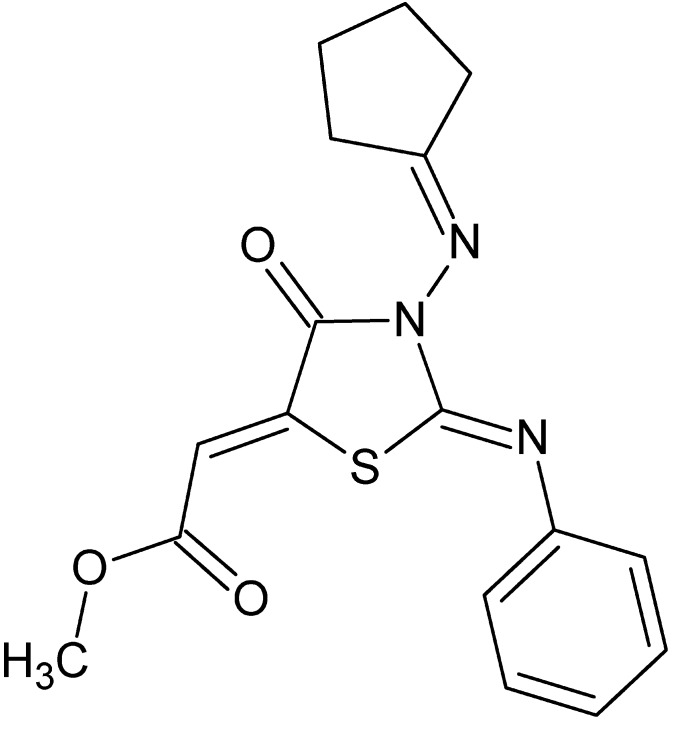


## Experimental   

### 

#### Crystal data  

C_17_H_17_N_3_O_3_S
*M* *_r_* = 343.41
Monoclinic, 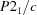

*a* = 9.9684 (2) Å
*b* = 9.9657 (2) Å
*c* = 16.9818 (3) Å
β = 105.9290 (6)°
*V* = 1622.23 (5) Å^3^
*Z* = 4
Cu *K*α radiation
μ = 1.96 mm^−1^
*T* = 100 K
0.17 × 0.16 × 0.09 mm

#### Data collection  

Bruker D8 VENTURE PHOTON 100 CMOS diffractometer
Absorption correction: multi-scan (*SADABS*; Bruker, 2013 ▶) *T*_min_ = 0.76, *T*_max_ = 0.84
17510 measured reflections
2951 independent reflections
2769 reflections with *I* > 2σ(*I*)
*R*_int_ = 0.023

#### Refinement  

*R*[*F* ^2^ > 2σ(*F* ^2^)] = 0.029
*wR*(*F* ^2^) = 0.075
*S* = 1.08
2951 reflections
218 parameters
61 restraints
H-atom parameters constrained
Δρ_max_ = 0.30 e Å^−3^
Δρ_min_ = −0.21 e Å^−3^

### 

Data collection: *APEX2* (Bruker, 2013 ▶); cell refinement: *SAINT* (Bruker, 2013 ▶); data reduction: *SAINT*; program(s) used to solve structure: *SHELXS2013* (Sheldrick, 2008 ▶); program(s) used to refine structure: *SHELXL2013* (Sheldrick, 2008 ▶); molecular graphics: *ORTEP-3 for Windows* (Farrugia, 2012 ▶); software used to prepare material for publication: *WinGX* (Farrugia, 2012 ▶) and *PLATON* (Spek, 2009 ▶).

## Supplementary Material

Crystal structure: contains datablock(s) global, I. DOI: 10.1107/S1600536814004048/gk2605sup1.cif

Structure factors: contains datablock(s) I. DOI: 10.1107/S1600536814004048/gk2605Isup2.hkl

Supporting information file. DOI: 10.1107/S1600536814004048/gk2605Isup3.cml

CCDC reference: 988094

Additional supporting information:  crystallographic information; 3D view; checkCIF report

## Figures and Tables

**Table 1:** Hydrogen-bond geometry (Å, °)

*D*—H⋯*A*	*D*—H	H⋯*A*	*D*⋯*A*	*D*—H⋯*A*
C10—H10*B*⋯O1^i^	0.99	2.57	3.2889 (17)	130
C11—H11*A*⋯O1^i^	0.99	2.58	3.2547 (16)	125

Symmetry code: (i) 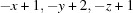.

**Fig. 1.**
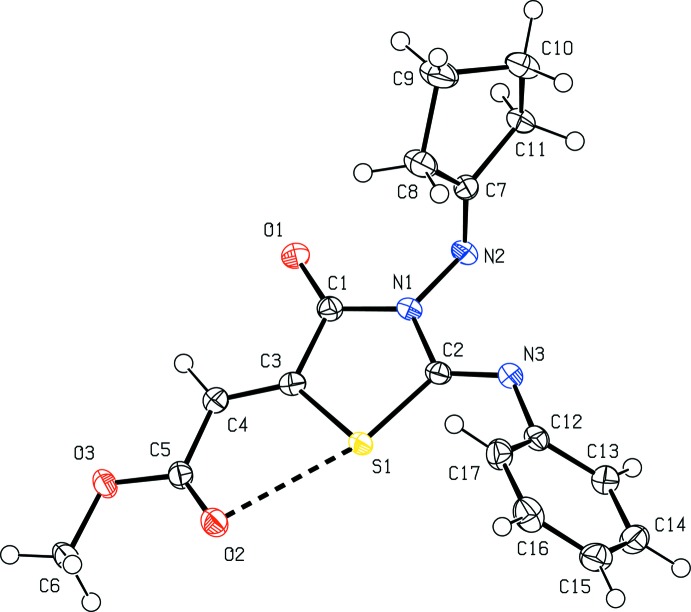
Perspective view of the title compound with 50% probability displacement ellipsoids.

**Fig. 2.**
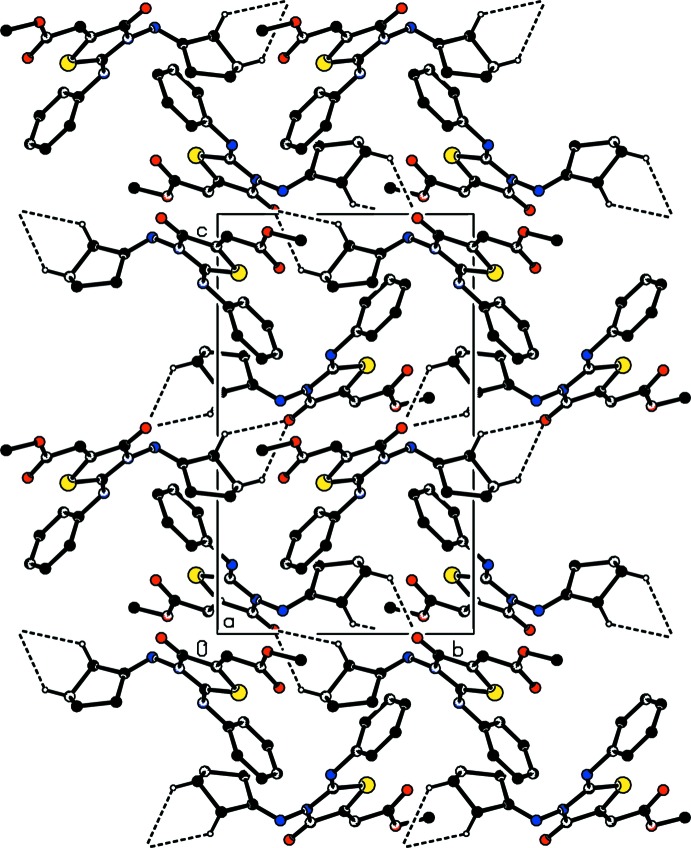
Crystal packing of the title compound viewed down the a axis. Only hydrogen atoms involved in C-H···O interactions (dashed lines) are shown.

## supplementary crystallographic information

### 1. Comment

Diversity in the biological response profile of thiazolidinone and analogous scaffolds has attracted much attention to the exploration of this skeleton for a variety of therapeutic applications. Thus, the successful pharmaceutical applications of pioglitazone as a hypoglycemic agent (Pfützner *et al*., 2007; Schianca *et al.*, 2012), thiazolidomycin activity against streptomyces species (Jain *et al.*, 2012), etozoline as an antihypertensive (Lant, 1986), and ralitoline as a potent anti-convulsant (Rock *et al.*, 1991) have established the wide spectrum potential of the thiazolidinone moiety. As part of our ongoing program of drug design and discovery, we report the synthesis and crystal structure of the title compound.

As shown in Fig. 1, the thiazole ring (S1/N1/C1–C3) of the title compound (I) is nearly planar with a maximum deviation of 0.015 (1) Å for N1. The cyclopentane ring (C7–C11) is twisted around the C9—C10 bond. The dihedral angle between the thiazole and phenyl rings is 53.84 (6)°. The C1–C3–C4–C5, C3–C4–C5–O2, C3–C4–C5–O3, C4–C5–O3–C6 and O2–C5–O3–C6 torsion angles are 178.90 (12), 6.7 (2), -174.42 (12), -178.11 (11) and 2.99 (19)°, respectively. All the bond lengths in (I) are comparable to those observed in similar structures (Akkurt *et al.*, 2009; Li *et al.*, 2011; Mague *et al.*, 2013; Mohamed *et al.*, 2013*a*,*b*; Pomés Hernández *et al.*, 1996; Sundar *et al.*, 2003).

Molecular conformation of (I) is stabilized by a short intramolecular (hypervalent) contact between the S1 atom and the ester group carbonyl O2 atom of 2.7931 (10) Å. In the crystal packing, the C—H···O interactions (Table 1, Fig. 2) with H···O distance of 2.57 - 2.58 Å generate chains of molecules propagating along the [110] direction. Furthermore, π-π stacking interactions [*Cg*1···*Cg*1 (1 - *x*, 1 - *y*, 1 - *z*) = 3.4677 (7) Å; where *Cg*1 is a centroid of the S1/N1/C1–C3 ring] between the thiazole rings organize these chains into the (001) layers.

### 2. Experimental

A solution of 1 mmol (233 mg) 2-cyclopentylidene-*N*-phenylhydrazinecarbothioamide in 15 ml ethanol was added dropwise to a solution of 1 mmol (142 mg) dimethyl but-2-ynedioate in 10 ml ethanol. The reaction mixture was stirred and refluxed at 351 K. The reaction progress was monitored by TLC until completion. On cooling a solid yellow product was precipitated, filtered off under vacuum and recrystallized from ethanol to furnish block-shaped yellow crystals (m.p. 541–543 K).

### 3. Refinement

All H atoms were positioned geometrically and treated as riding atoms, with C—H = 0.95 Å (aromatic H), 0.98 Å (methyl H) and 0.99 Å (methylene H), with *U*_iso_(H) = 1.5 *U*_iso_(C) for methyl H atoms and *U*_iso_(H) = 1.2 *U*_iso_(C) for the others. The components of the displacement parameters in the direction of the bond between non-hydrogen atoms were restrained to be equal within an effective standard deviation of 0.01 (DELU instruction).

### Crystal data

**Table:**

C_17_H_17_N_3_O_3_S	*F*(000) = 720
*M**_r_* = 343.41	*D*_x_ = 1.406 Mg m^−^^3^
Monoclinic, *P*2_1_/*c*	Cu *K*α radiation, λ = 1.54178 Å
Hall symbol: -P 2ybc	Cell parameters from 9943 reflections
*a* = 9.9684 (2) Å	θ = 4.4–68.3°
*b* = 9.9657 (2) Å	µ = 1.96 mm^−^^1^
*c* = 16.9818 (3) Å	*T* = 100 K
β = 105.9290 (6)°	Block, yellow
*V* = 1622.23 (5) Å^3^	0.17 × 0.16 × 0.09 mm
*Z* = 4	

### Data collection

**Table:**

Bruker D8 VENTURE PHOTON 100 CMOS diffractometer	2951 independent reflections
Radiation source: INCOATEC IµS micro-focus source	2769 reflections with *I* > 2σ(*I*)
Mirror monochromator	*R*_int_ = 0.023
Detector resolution: 10.4167 pixels mm^-1^	θ_max_ = 68.2°, θ_min_ = 4.6°
ω scans	*h* = −12→12
Absorption correction: multi-scan (*SADABS*; Bruker, 2013)	*k* = −11→12
*T*_min_ = 0.76, *T*_max_ = 0.84	*l* = −20→20
17510 measured reflections	

### Refinement

**Table:**

Refinement on *F*^2^	61 restraints
Least-squares matrix: full	Hydrogen site location: inferred from neighbouring sites
*R*[*F*^2^ > 2σ(*F*^2^)] = 0.029	H-atom parameters constrained
*wR*(*F*^2^) = 0.075	*w* = 1/[σ^2^(*F*_o_^2^) + (0.038*P*)^2^ + 0.6771*P*] where *P* = (*F*_o_^2^ + 2*F*_c_^2^)/3
*S* = 1.08	(Δ/σ)_max_ < 0.001
2951 reflections	Δρ_max_ = 0.30 e Å^−^^3^
218 parameters	Δρ_min_ = −0.21 e Å^−^^3^

### Special details

**Table:**

Geometry. Bond distances, angles *etc*. have been calculated using the rounded fractional coordinates. All su's are estimated from the variances of the (full) variance-covariance matrix. The cell e.s.d.'s are taken into account in the estimation of distances, angles and torsion angles
Refinement. Refinement on *F*^2^ for ALL reflections except those flagged by the user for potential systematic errors. Weighted *R*-factors *wR* and all goodnesses of fit *S* are based on *F*^2^, conventional *R*-factors *R* are based on *F*, with *F* set to zero for negative *F*^2^. The observed criterion of *F*^2^ > σ(*F*^2^) is used only for calculating -*R*-factor-obs *etc*. and is not relevant to the choice of reflections for refinement. *R*-factors based on *F*^2^ are statistically about twice as large as those based on *F*, and *R*-factors based on ALL data will be even larger.

### Fractional atomic coordinates and isotropic or equivalent isotropic displacement parameters (Å^2^)

**Table:**

	*x*	*y*	*z*	*U*_iso_*/*U*_eq_	
S1	0.49073 (3)	0.41667 (3)	0.35998 (2)	0.0187 (1)	
O1	0.66547 (10)	0.71951 (9)	0.49052 (6)	0.0244 (3)	
O2	0.72709 (10)	0.25889 (9)	0.37200 (6)	0.0258 (3)	
O3	0.93931 (9)	0.30447 (9)	0.45742 (6)	0.0238 (3)	
N1	0.44614 (11)	0.65036 (10)	0.41830 (6)	0.0190 (3)	
N2	0.36915 (11)	0.75447 (11)	0.44449 (7)	0.0214 (3)	
N3	0.24379 (11)	0.55652 (11)	0.33548 (7)	0.0204 (3)	
C1	0.58785 (13)	0.63691 (13)	0.44889 (8)	0.0193 (3)	
C2	0.37387 (13)	0.54855 (12)	0.36801 (8)	0.0184 (3)	
C3	0.63174 (13)	0.50466 (13)	0.42232 (7)	0.0186 (3)	
C4	0.76427 (13)	0.46352 (13)	0.44650 (8)	0.0202 (4)	
C5	0.80432 (13)	0.33216 (13)	0.42077 (8)	0.0207 (4)	
C6	0.98665 (15)	0.17461 (14)	0.43752 (9)	0.0276 (4)	
C7	0.36894 (13)	0.86381 (13)	0.40585 (8)	0.0202 (4)	
C8	0.43371 (17)	0.89572 (14)	0.33790 (9)	0.0298 (4)	
C9	0.41616 (18)	1.04928 (15)	0.32757 (11)	0.0364 (5)	
C10	0.28241 (16)	1.07780 (14)	0.35110 (9)	0.0300 (4)	
C11	0.29241 (14)	0.98521 (13)	0.42434 (8)	0.0241 (4)	
C12	0.17495 (13)	0.45669 (13)	0.27910 (8)	0.0209 (3)	
C13	0.05398 (14)	0.39885 (14)	0.28986 (9)	0.0249 (4)	
C14	−0.01893 (15)	0.30450 (15)	0.23424 (9)	0.0283 (4)	
C15	0.02709 (15)	0.26783 (14)	0.16740 (9)	0.0291 (4)	
C16	0.14570 (15)	0.32725 (16)	0.15559 (9)	0.0300 (4)	
C17	0.21879 (14)	0.42310 (14)	0.21052 (8)	0.0252 (4)	
H4	0.83320	0.51970	0.48060	0.0240*	
H6A	0.94260	0.10380	0.46180	0.0410*	
H6B	1.08820	0.16880	0.45940	0.0410*	
H6C	0.96120	0.16350	0.37790	0.0410*	
H8A	0.38460	0.84850	0.28680	0.0360*	
H8B	0.53350	0.87020	0.35310	0.0360*	
H9A	0.49630	1.09710	0.36430	0.0440*	
H9B	0.40730	1.07640	0.27030	0.0440*	
H10A	0.19940	1.05620	0.30550	0.0360*	
H10B	0.27760	1.17310	0.36660	0.0360*	
H11A	0.34520	1.02850	0.47600	0.0290*	
H11B	0.19850	0.96050	0.42870	0.0290*	
H13	0.02130	0.42400	0.33530	0.0300*	
H14	−0.10100	0.26480	0.24210	0.0340*	
H15	−0.02240	0.20230	0.12990	0.0350*	
H16	0.17740	0.30250	0.10970	0.0360*	
H17	0.29840	0.46550	0.20120	0.0300*	

### Atomic displacement parameters (Å^2^)

**Table:**

	*U*^11^	*U*^22^	*U*^33^	*U*^12^	*U*^13^	*U*^23^
S1	0.0170 (2)	0.0147 (2)	0.0233 (2)	0.0017 (1)	0.0036 (1)	−0.0023 (1)
O1	0.0223 (5)	0.0191 (5)	0.0305 (5)	−0.0023 (4)	0.0053 (4)	−0.0060 (4)
O2	0.0216 (5)	0.0233 (5)	0.0304 (5)	0.0028 (4)	0.0037 (4)	−0.0056 (4)
O3	0.0179 (5)	0.0226 (5)	0.0294 (5)	0.0051 (4)	0.0040 (4)	−0.0028 (4)
N1	0.0194 (5)	0.0141 (5)	0.0238 (5)	0.0017 (4)	0.0063 (4)	−0.0014 (4)
N2	0.0222 (6)	0.0160 (5)	0.0274 (6)	0.0036 (4)	0.0092 (4)	−0.0026 (4)
N3	0.0181 (5)	0.0181 (5)	0.0245 (6)	0.0016 (4)	0.0051 (4)	0.0009 (4)
C1	0.0200 (6)	0.0176 (6)	0.0211 (6)	0.0003 (5)	0.0072 (5)	0.0012 (5)
C2	0.0210 (6)	0.0146 (6)	0.0206 (6)	0.0016 (5)	0.0076 (5)	0.0014 (5)
C3	0.0210 (6)	0.0161 (6)	0.0191 (6)	−0.0013 (5)	0.0063 (5)	0.0000 (5)
C4	0.0188 (6)	0.0193 (7)	0.0218 (6)	−0.0009 (5)	0.0043 (5)	−0.0006 (5)
C5	0.0178 (6)	0.0219 (7)	0.0223 (6)	0.0016 (5)	0.0054 (5)	0.0016 (5)
C6	0.0247 (7)	0.0240 (7)	0.0329 (7)	0.0085 (6)	0.0060 (6)	−0.0022 (6)
C7	0.0170 (6)	0.0182 (7)	0.0239 (6)	−0.0004 (5)	0.0032 (5)	−0.0041 (5)
C8	0.0371 (8)	0.0209 (7)	0.0371 (8)	0.0065 (6)	0.0197 (7)	0.0048 (6)
C9	0.0487 (10)	0.0221 (8)	0.0451 (9)	0.0068 (7)	0.0243 (8)	0.0076 (7)
C10	0.0359 (8)	0.0185 (7)	0.0347 (8)	0.0058 (6)	0.0084 (6)	0.0005 (6)
C11	0.0253 (7)	0.0172 (7)	0.0302 (7)	0.0034 (5)	0.0084 (5)	−0.0032 (5)
C12	0.0180 (6)	0.0174 (6)	0.0247 (6)	0.0037 (5)	0.0015 (5)	0.0024 (5)
C13	0.0220 (7)	0.0241 (7)	0.0281 (7)	0.0000 (5)	0.0062 (6)	0.0009 (6)
C14	0.0235 (7)	0.0249 (7)	0.0336 (8)	−0.0032 (6)	0.0029 (6)	0.0028 (6)
C15	0.0286 (7)	0.0230 (7)	0.0295 (7)	−0.0002 (6)	−0.0025 (6)	−0.0017 (6)
C16	0.0280 (7)	0.0351 (8)	0.0242 (7)	0.0060 (6)	0.0024 (6)	−0.0035 (6)
C17	0.0200 (7)	0.0279 (7)	0.0262 (7)	0.0016 (5)	0.0038 (5)	0.0005 (6)

### Geometric parameters (Å, º)

**Table:**

S1—C2	1.7861 (13)	C13—C14	1.388 (2)
S1—C3	1.7461 (13)	C14—C15	1.385 (2)
O1—C1	1.2148 (16)	C15—C16	1.385 (2)
O2—C5	1.2091 (16)	C16—C17	1.393 (2)
O3—C5	1.3471 (16)	C4—H4	0.9500
O3—C6	1.4489 (17)	C6—H6A	0.9800
N1—N2	1.4320 (15)	C6—H6B	0.9800
N1—C1	1.3713 (17)	C6—H6C	0.9800
N1—C2	1.3929 (16)	C8—H8A	0.9900
N2—C7	1.2717 (17)	C8—H8B	0.9900
N3—C2	1.2648 (18)	C9—H9A	0.9900
N3—C12	1.4201 (17)	C9—H9B	0.9900
C1—C3	1.4968 (18)	C10—H10A	0.9900
C3—C4	1.3358 (19)	C10—H10B	0.9900
C4—C5	1.4696 (18)	C11—H11A	0.9900
C7—C8	1.503 (2)	C11—H11B	0.9900
C7—C11	1.5086 (19)	C13—H13	0.9500
C8—C9	1.545 (2)	C14—H14	0.9500
C9—C10	1.520 (2)	C15—H15	0.9500
C10—C11	1.530 (2)	C16—H16	0.9500
C12—C13	1.393 (2)	C17—H17	0.9500
C12—C17	1.3924 (19)		
			
C2—S1—C3	91.01 (6)	O3—C6—H6A	109.00
C5—O3—C6	115.04 (10)	O3—C6—H6B	109.00
N2—N1—C1	122.50 (10)	O3—C6—H6C	109.00
N2—N1—C2	119.15 (11)	H6A—C6—H6B	109.00
C1—N1—C2	117.82 (11)	H6A—C6—H6C	110.00
N1—N2—C7	112.69 (11)	H6B—C6—H6C	109.00
C2—N3—C12	119.89 (11)	C7—C8—H8A	111.00
O1—C1—N1	125.42 (12)	C7—C8—H8B	111.00
O1—C1—C3	125.44 (12)	C9—C8—H8A	111.00
N1—C1—C3	109.14 (11)	C9—C8—H8B	111.00
S1—C2—N1	110.15 (9)	H8A—C8—H8B	109.00
S1—C2—N3	128.67 (10)	C8—C9—H9A	111.00
N1—C2—N3	121.19 (12)	C8—C9—H9B	111.00
S1—C3—C1	111.82 (9)	C10—C9—H9A	111.00
S1—C3—C4	126.58 (10)	C10—C9—H9B	111.00
C1—C3—C4	121.59 (12)	H9A—C9—H9B	109.00
C3—C4—C5	120.56 (12)	C9—C10—H10A	111.00
O2—C5—O3	124.08 (12)	C9—C10—H10B	111.00
O2—C5—C4	124.60 (12)	C11—C10—H10A	111.00
O3—C5—C4	111.32 (11)	C11—C10—H10B	111.00
N2—C7—C8	129.59 (12)	H10A—C10—H10B	109.00
N2—C7—C11	120.60 (12)	C7—C11—H11A	111.00
C8—C7—C11	109.79 (11)	C7—C11—H11B	111.00
C7—C8—C9	103.75 (12)	C10—C11—H11A	111.00
C8—C9—C10	103.62 (13)	C10—C11—H11B	111.00
C9—C10—C11	103.58 (12)	H11A—C11—H11B	109.00
C7—C11—C10	103.79 (11)	C12—C13—H13	120.00
N3—C12—C13	118.49 (12)	C14—C13—H13	120.00
N3—C12—C17	121.86 (12)	C13—C14—H14	120.00
C13—C12—C17	119.47 (12)	C15—C14—H14	120.00
C12—C13—C14	120.09 (13)	C14—C15—H15	120.00
C13—C14—C15	120.46 (14)	C16—C15—H15	120.00
C14—C15—C16	119.58 (14)	C15—C16—H16	120.00
C15—C16—C17	120.43 (14)	C17—C16—H16	120.00
C12—C17—C16	119.90 (13)	C12—C17—H17	120.00
C3—C4—H4	120.00	C16—C17—H17	120.00
C5—C4—H4	120.00		
			
C3—S1—C2—N1	−1.19 (9)	O1—C1—C3—C4	3.2 (2)
C3—S1—C2—N3	179.13 (13)	N1—C1—C3—S1	1.81 (13)
C2—S1—C3—C1	−0.35 (10)	N1—C1—C3—C4	−177.24 (12)
C2—S1—C3—C4	178.65 (12)	S1—C3—C4—C5	0.00 (19)
C6—O3—C5—O2	−2.99 (19)	C1—C3—C4—C5	178.90 (12)
C6—O3—C5—C4	178.11 (11)	C3—C4—C5—O2	6.7 (2)
C1—N1—N2—C7	84.66 (15)	C3—C4—C5—O3	−174.42 (12)
C2—N1—N2—C7	−103.83 (13)	N2—C7—C8—C9	−171.37 (15)
N2—N1—C1—O1	−11.7 (2)	C11—C7—C8—C9	10.31 (16)
N2—N1—C1—C3	168.71 (10)	N2—C7—C11—C10	−164.51 (13)
C2—N1—C1—O1	176.71 (13)	C8—C7—C11—C10	14.00 (15)
C2—N1—C1—C3	−2.90 (15)	C7—C8—C9—C10	−30.79 (16)
N2—N1—C2—S1	−169.23 (8)	C8—C9—C10—C11	39.85 (15)
N2—N1—C2—N3	10.49 (18)	C9—C10—C11—C7	−33.10 (14)
C1—N1—C2—S1	2.68 (14)	N3—C12—C13—C14	177.64 (13)
C1—N1—C2—N3	−177.61 (12)	C17—C12—C13—C14	2.6 (2)
N1—N2—C7—C8	1.4 (2)	N3—C12—C17—C16	−178.17 (13)
N1—N2—C7—C11	179.58 (11)	C13—C12—C17—C16	−3.2 (2)
C12—N3—C2—S1	−5.62 (19)	C12—C13—C14—C15	−0.5 (2)
C12—N3—C2—N1	174.72 (11)	C13—C14—C15—C16	−0.9 (2)
C2—N3—C12—C13	130.97 (14)	C14—C15—C16—C17	0.2 (2)
C2—N3—C12—C17	−54.06 (18)	C15—C16—C17—C12	1.9 (2)
O1—C1—C3—S1	−177.80 (11)		

### Hydrogen-bond geometry (Å, º)

**Table:**

*D*—H···*A*	*D*—H	H···*A*	*D*···*A*	*D*—H···*A*
C10—H10*B*···O1^i^	0.99	2.57	3.2889 (17)	130
C11—H11*A*···O1^i^	0.99	2.58	3.2547 (16)	125

Symmetry code: (i) −*x*+1, −*y*+2, −*z*+1.
